# Case report: Giant atypical granular cell tumor of the median nerve

**DOI:** 10.3389/fneur.2023.1221912

**Published:** 2023-09-29

**Authors:** Jun-Peng Liu, Ling-Xie Song, Zi-Yu Xu, Yue Wu, Xing-Chen Yao, Meng Li, Xin-Ru Du

**Affiliations:** Department of Orthopaedic Surgery and Pathology, Beijing Chaoyang Hospital, Capital Medical University, Beijing, China

**Keywords:** granular cell tumor, median nerve, pathological diagnosis, immunohistochemistry, case report

## Abstract

Granular cell tumors are extremely uncommon soft tissue neoplasms that mostly occur in the head and neck regions. Granular cell tumors are generally benign, asymptomatic, and rarely involve the median nerve. Due to the lack of awareness about granular cell tumors, they are easily misdiagnosed and mistreated in primary hospitals. Here, we report a giant atypical granular cell tumor located on the median nerve, approximately 12 cm in size, with unusual symptoms of median nerve damage. Magnetic resonance imaging revealed a fusiform mass that was hyperintense on T_2_-weighted images and iso-hypointense on T_1_-weighted images. The mass was subsequently biopsied and found to be a granular cell tumor. The tumor was resected, and a pathological examination was performed. Pathological examination revealed necrotic foci, abundant eosinophilic granules, pustular ovoid bodies, and multiple mitoses. Immunohistochemical staining revealed that the tumor cells were positive for S-100, CD68, SMA, SOX-10, Calretinin, and TFE3. The integrated diagnosis was an atypical granular cell tumor. To the best of our knowledge, this is the first report of an atypical granular cell tumor involving the median nerve. Furthermore, we comprehensively reviewed the existing literature to provide a concise summary of the diagnostic criteria, imaging findings, and pathological features of granular cell tumors. Given the high recurrence and metastasis rates of this disease, granular cell tumors of the median nerve should be considered when a patient presents with symptoms of median nerve impairment. The diagnosis of atypical granular cell tumors relies on pathological examination. In addition, extensive resection and long-term follow-up are necessary to improve prognosis.

## Introduction

Granular cell tumors (GCT) are rare soft tissue tumors derived from Schwann cells (1) and account for only 0.5% of all soft tissue tumors (2). Of these, approximately 98% are benign (3). Malignant atypical GCTs are extremely rare (4–6). GCTs are more common in individuals 40–60 years old, with a male-to-female ratio of 1:1.8–2.9 (7). It can occur anywhere in the body, often in the head and neck, especially the tongue (8), and rarely in the peripheral nerves (3, 9). To date, only three cases of GCT involving the median nerve and its branches have been reported (Table 1) (10–12). Herein, we report a case of a giant atypical GCT of the median nerve. To the best of our knowledge, this is the first report of an atypical granular cell tumor involving the median nerve. This is the largest and most typical case of GCT involving the median nerve reported in the current literature, with a maximum diameter of approximately 12 cm.

**Table 1:** Literature review of granular cell tumors of the median nerve.

**References**	**Age/sex**	**Location**	**Nature**	**Size (cm)**	**Duration**	**Symptoms**	**Treatment**	**Follow-up (months)**
Condit et al. (10)	25/F	Volar right wrist	Benign	0.5 × 0.5	1 year	Mass, pain, paresthesia	Excision	12-NR
Tsukamoto et al. (11)	67/F	Right palm	Malignant	5.2 × 5 × 4	6 years	Mass	Excision	5-R
Joshi et al. (12)	48/F	Right upper arm	Benign	9 × 3 × 2	15 years	Mass, pain, paresthesia	Excision	13-NR
Present case	59/F	Right forearm	Atypical	12.5 × 2.2 × 3.5	1 year	Mass, pain, paresthesia	Excision	2-NR

F, female; NR, no recurrence; R, recurrence.

## Case presentation

A 59-year-old woman presented with a progressively enlarged mass on her right forearm that had persisted for more than 1 year. In the past 6 months, the mass had increased significantly, accompanied by numbness and pain in the right palm, as well as numbness alone in three and a half fingers on the radial side. In the past 1 month, the patient had developed gradually worsening pain in her right wrist. She visited our hospital on 8 February 2023. The patient denied any history of trauma or disease at the lesion site and had no family history of GCT. Physical examination revealed a fusiform mass measuring approximately 8 × 2 cm between the flexor carpi radialis and palmaris longus of the right forearm. The patient's skin was tough and slightly raised. The tumor was fixed in a deep structure, did not adhere to the skin, and could move with wrist movement. When percussing the tumor, discharge-like numbness was observed in the distal end of the index and middle fingers. The right thenar muscles showed significant atrophy. There were no lesions in organs other than the right forearm.

**Figure 1:**
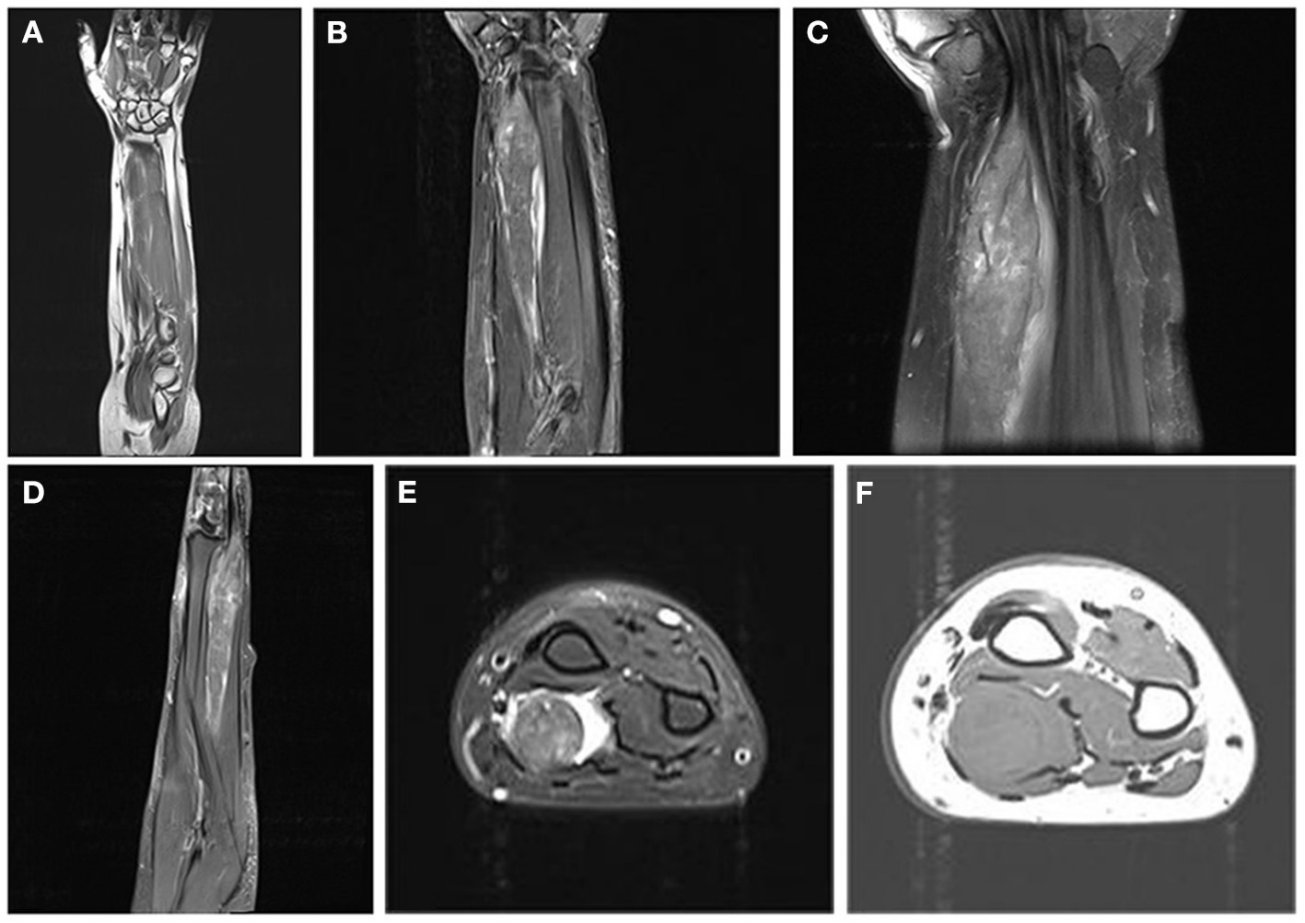
Magnetic resonance images. **(A)** Coronal T_1_-weighted images show that the mass is strip-shaped and iso-hypointense. **(B)** Coronal T_2_-weighted images show heterogeneous high intensity. **(C)** Coronal proton density-weighted fat-suppressed images show a spindle-shaped tumor with clear borders. **(D)** Sagittal T_2_-weighted image. **(E)** Axial fat-suppressed T_2_-weighted images show heterogeneous density inside the tumor and high intensity around the tumor. **(F)** Axial T_1_-weighted image.

Magnetic resonance images (MRI) of the right forearm showed iso-hypointense on T_1_-weighted images (Figure 1A) and hyperintensity on T_2_-weighted images (Figure 1B). The tumor was a 1.9 × 1.8 × 7.5 cm fusiform mass. Adjacent tendons were compressed and displaced (Figures 1C, D). On axial images, the tumor was surrounded by high signal intensity, and there was a heterogeneous signal area in the tumor (Figures 1E, F).

**Figure 2:**
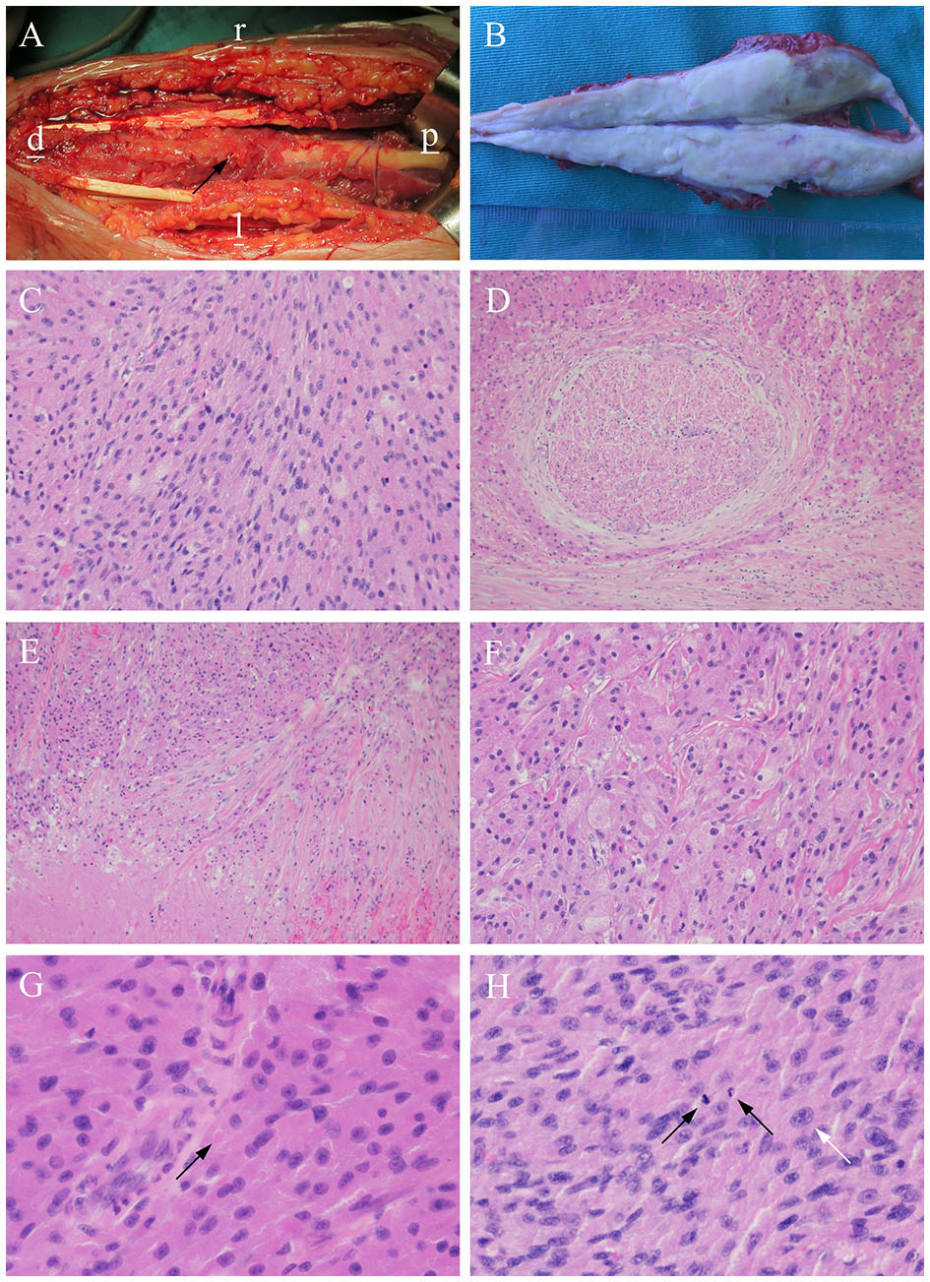
Histopathological features of the tumor. **(A)** It is closely related to the surrounding muscles and manifests as a fusiform bulge on the median nerve (arrow) (Note: l: left; r: right; d: distal; p: proximal). **(B)** The tumor is dissected, and the surface is uniformly gray-white. **(C)** The tumor cells are arranged in sheets, and cords of plump [hematoxylin and eosin (H&E) stain, × 200]. **(D)** Entrapped nerve fibers surrounded by tumor (H&E stain, × 100). **(E)** Necrosis foci (H&E stain, × 100). **(F)** Granular and intensely eosinophilic cytoplasm and some cytoplasms are vacuolated (H&E stain, × 200). **(G)** Pustular ovoid body (arrow) (H&E stain, × 400). **(H)** Tumor cells with multiple mitoses (2/10 HPF) (black arrows) and pleomorphism (white arrow) (H&E stain, × 400).

A peripheral nerve sheath tumor was suspected. Due to the lack of typical imaging signs, an incisional biopsy was performed. Biopsy results indicated a GCT. The patient underwent surgical resection 1 week after the biopsy. Intraoperatively, we observed that the tumor infiltrated a 12 cm segment of the median nerve between the superficial and deep flexor digitorums. The tumor appeared fusiform, grayish-white, tough, with ill-defined borders, and was unencapsulated. The two ends of the tumor grew continuously and had no boundaries with the nerves (Figure 2A). The tumor and adjacent muscle tissue were completely resected, and a pathological examination was performed.

Macroscopically, a gray-white fusiform mass measuring 12.5 × 2.2 × 3.5 cm (Figure 2B) was non-encapsulated, with the muscle attached to the surface. The tumor was tough and solid with no obvious fluctuations or necrosis. The surface of the incision was grayish-white or grayish-yellow and partially grayish-red, with normal margins.

Histopathologically, the tumor was arranged as sheets and cords of plump polygonal cells with abundant eosinophilic granular cytoplasm (Figure 2C). Entrapped nerve fibers were also identified in the tumors (Figure 2D). In addition, necrosis was observed in some areas (Figure 2E). A small portion of the cytoplasm was vacuolated (Figure 2F), and oval eosinophilic bodies surrounded by halos were occasionally observed (Figure 2G). The tumor showed atypical features such as large nuclei with prominent nucleoli, a high nuclear:cytoplasmic ratio, pleomorphism, and multiple mitoses (two mitotic counts per 10 high-power fields) (Figure 2H).

**Figure 3:**
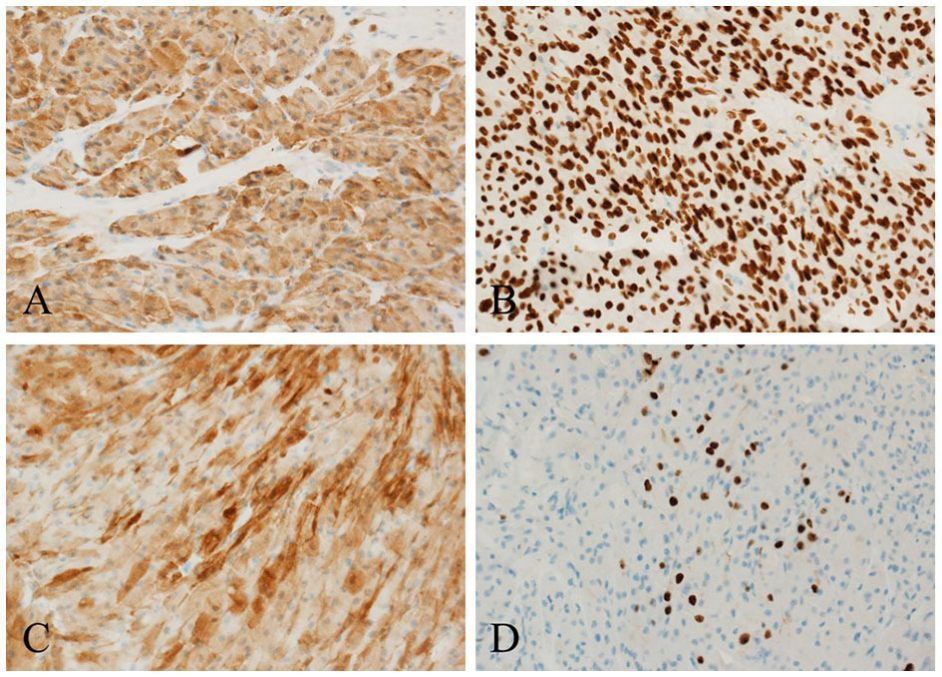
Histochemical staining. The tumor cells are strongly positive for **(A)** S-100, **(B)** SOX10, and **(C)** Calretinin, and the **(D)** Ki-67 labeling index is 10% ( × 200).

Immunohistochemically, the tumor cells were positive for S-100 (Figure 3A), CD68, SMA, SOX-10 (Figure 3B), Calretinin (Figure 3C), and TFE3. The Ki-67 labeling index was 10% (Figure 3D). The pathological diagnosis was an atypical GCT of the median nerve (13).

Post-operatively, the distal interphalangeal joints of the thumb and index finger were weakly flexed, the remaining fingers moved well, and the three-and-a-half fingers on the radial side were numb. The thumb opposition function was poor. After 2 months of operation, the numbness of the patient's fingers improved, and muscle strength was enhanced. The patient and her family were satisfied with the effects of the treatment. No signs of recurrence have been observed.

## Discussion

GCTs, rare benign neoplasms, were first reported by Abrikossoff and Myome (14). GCTs generally have no obvious etiology (15), which is consistent with this case. The clinical presentation of GCTs involving the nerves depends on the location of the tumor and its relationship with the peripheral nerves. However, because of its small diameter (average, 2.1 cm) (7) and slow growth, the mass is often asymptomatic (4). In the present case, the maximum diameter of the tumor was 12 cm, and the scope and degree of nerve invasion were severe; therefore, the patient presented with symptoms of median nerve damage. Peripheral nerve tumors are primarily neurofibromas or schwannomas. GCTs are relatively rare and can easily be misdiagnosed. Therefore, the possibility of GCTs should be seriously considered when diagnosing forearm tumors with symptoms of pure median nerve injury. GCT can be initially differentiated from other tumors using MRI, and the degree of malignancy can be determined using biopsy.

Currently, most scholars accept the diagnostic criteria of Fanburg-Smith (F-S) and believe that the diagnosis of malignant granular cell tumor (MGCT) needs to consider the following aspects: necrosis, multiple spindle cells, vesicular nuclei with large nucleoli, an increased mitotic rate (>2 mitoses/10 high-power fields at × 200 magnification), high nuclear-to-cytoplasmic ratio, and pleomorphism (13). Based on these criteria, GCT can be roughly classified into three categories: benign (no criteria or focal pleomorphism), atypical (criteria 1 to 2), and malignant (criteria 3 to 6). However, as the number of cases was limited, the pathological criteria were imperfect. Therefore, it is necessary to incorporate the clinical behavior of the tumor into diagnostic criteria. Gamboa divided MGCT into two groups: Gamboa type I, with benign histology and malignant behavior, and Gamboa type II, with malignant histology and clinical manifestations (16). Notably, locally invasive growth and a close relationship with the nerve can also be observed in benign GCT (17) that are not malignant. Some scholars (18) recommend combining the Gamboa classification with the F-S criteria for clinical diagnosis. If the tumor has metastasized, it should be diagnosed as malignant even if the histology is benign. However, for cases without metastasis, a diagnosis should be made according to the F-S criteria. Based on the above criteria, the patient was diagnosed with an atypical GCT. In addition, tumors with rapid growth, recurrence (4), diameter > 5 cm (19, 20), Ki-67 level > 10%, or P53 > 50% usually indicate that the disease is prone to progression and poor prognosis (5, 13, 21–23). These factors should be labeled to guide treatment and assist in determining patient prognosis.

GCTs are usually iso-hypointense on T_1_WI and slightly higher than muscle but lower than fat on T_2_WI (24). In this case, the tumor was surrounded by hyperintensity. In general soft tissue tumors, this phenomenon usually indicates that the tumor is malignant (25). However, for GCT, peripheral hyperintensities may be due to compression of the surrounding tissues or severe inflammation with lymphocyte infiltration around the tumor rather than specific signs suggestive of malignancy (24, 26). The tumor center presents as a mixed low-density area on MRI, which may be associated with the fibrous component observed among the tumor cells in the pathological slices (24, 27). This distinctive feature of a low-signal zone on MRI is not typically found in other soft tissue tumors and can help identify them.

Histopathological examination is the gold standard for the diagnosis of GCTs (25). Tumor cells are usually arranged in sheets or cords, which was consistent with this case. Its typical feature is rich granular eosinophilic cytoplasm inside the tumor cells. In addition, we observed the formation of oval bodies with surrounding halo zones that are called pustule–ovoid bodies of Milian (28). This is a characteristic pathological change observed in GCT (5, 28).

In our case, the immunohistochemical examination was positive for S-100, SOX10, TFE3, Calretinin, SMA, and CD68. Diffuse positivity for S-100 proteins is a specific finding in GCT that supports the neural origin of GCT (29). Aegani et al. (30) first discovered the expression of TFE3 in GCT, with a positive expression rate of 91% (31). This particular staining may be due to the impairment of ATP6AP1 and ATP6AP2, resulting in the impairment of V-ATPase (H+ ATPase), which activates the lysosomal repression-inducible transcription factor TFE3 (32). Its expression reflects an increase in the number of secondary lysosomes in tumor cells (33), which exhibit abundant eosinophilic granules. Ki-67, a proliferation-related nuclear protein, is expressed during the proliferation cycle (non-G0 phase) of all cells. Its expression is correlated with the degree of malignancy and poor outcomes in GCT. The Ki-67 index of benign GCT is usually only 1–2% (23), whereas that of MGCT is usually >20% (22). The Ki-67 index in this patient was approximately 10%, which was consistent with an atypical GCT (5, 13, 23).

Some scholars believe that for tumors with a diameter of >4 cm located in sites prone to metastasis [regional lymph nodes, lungs, liver, and bone (5, 34)], or rapidly growing in a short period, the possibility of malignancy should be fully considered, and extended resection should be performed. According to the cases reported by Fanburg-Smith et al. and Kapur et al. (13, 23), atypical GCTs seldom metastasize. However, the local recurrence rate is high, and there have been cases of malignant transformation of GCT (35). Therefore, long-term follow-up is essential.

## Conclusion

In conclusion, we report a rare case of giant atypical GCT of the median nerve. The possibility of GCT should be seriously considered when diagnosing forearm tumors with pure median nerve injury symptoms. Extensive resection and long-term follow-up are necessary. Given the rarity of such tumors, their pathogenesis, imaging findings, histopathological features, and prognosis still require future studies.

## Author's note

The authors have read the CARE Checklist (2013), and the manuscript was prepared and revised according to the CARE Checklist (2013).

## Data availability statement

The original contributions presented in the study are included in the article/supplementary material, further inquiries can be directed to the corresponding author.

## Ethics statement

Written informed consent was obtained from the individual for the publication of any potentially identifiable images or data included in this article.

## Author contributions

J-PL, L-XS, ML, and YW contributed to the data collection and wrote the first draft of the manuscript. L-XS and J-PL designed the figures and table. X-RD, X-CY, Z-YX, and YW critically revised the manuscript. All authors have read and approved the submitted version.
